# Inflammasome activation by viral infection: mechanisms of activation and regulation

**DOI:** 10.3389/fmicb.2023.1247377

**Published:** 2023-08-07

**Authors:** Wen Shi, Mengyun Jin, Hao Chen, Zongxue Wu, Liuyang Yuan, Si Liang, Xiaohan Wang, Fareed Uddin Memon, Fatma Eldemery, Hongbin Si, Changbo Ou

**Affiliations:** ^1^College of Animal Science and Technology, Guangxi University, Nanning, China; ^2^QYH Biotech Ltd., Beijing, China; ^3^Department of Hygiene and Zoonoses, Faculty of Veterinary Medicine, Mansoura University, Mansoura, Egypt; ^4^Guangxi Zhuang Autonomous Region Engineering Research Center of Veterinary Biologics, Nanning, China; ^5^Guangxi Key Laboratory of Animal Reproduction, Breeding and Disease Control, Nanning, China

**Keywords:** Inflammasome, virus infection, inflammatory response, NLRP, regulation

## Abstract

Viral diseases are the most common problems threatening human health, livestock, and poultry industries worldwide. Viral infection is a complex and competitive dynamic biological process between a virus and a host/target cell. During viral infection, inflammasomes play important roles in the host and confer defense mechanisms against the virus. Inflammasomes are polymeric protein complexes and are considered important components of the innate immune system. These immune factors recognize the signals of cell damage or pathogenic microbial infection after activation by the canonical pathway or non-canonical pathway and transmit signals to the immune system to initiate the inflammatory responses. However, some viruses inhibit the activation of the inflammasomes in order to replicate and proliferate in the host. In recent years, the role of inflammasome activation and/or inhibition during viral infection has been increasingly recognized. Therefore, in this review, we describe the biological properties of the inflammasome associated with viral infection, discuss the potential mechanisms that activate and/or inhibit NLRP1, NLRP3, and AIM2 inflammasomes by different viruses, and summarize the reciprocal regulatory effects of viral infection on the NLRP3 inflammasome in order to explore the relationship between viral infection and inflammasomes. This review will pave the way for future studies on the activation mechanisms of inflammasomes and provide novel insights for the development of antiviral therapies.

## 1. Introduction

When the host immune system is weakened, microorganisms compete to invade it. The innate immune system is the first-line host defense mechanism against microorganisms. Once the host is infected by a virus, the innate immune system is activated and initiates a series of germline-encoded pattern recognition receptors (PRRs) to recognize microbe-derived pathogen-associated molecular patterns (PAMPs, a conserved microbial structure) and endogenous damage-associated molecular patterns (DAMPs, a marker of tissue damage generated by the host cell) (Medzhitov and Janeway, 2000). A wide variety of PRRs have been studied, such as toll-like receptors (TLRs), nucleotide oligomerization domain (NOD)-like receptors (NLRs), RIG-I-like receptors (RLRs), C-type lectin receptors (CLRs), and absent in melanoma 2 (AIM2)-like receptors (ALRs) (Franchi et al., 2009; Brubaker et al., 2015; Kumari et al., 2020). The intracellular signaling cascades triggered by these PRRs lead to post-transcriptional regulation of cytokine release and cell death (Takeuchi and Akira, 2010). Activation of these receptors by the virus then sends signals inside the organism to take a series of actions, such as inflammasome-mediated inflammatory responses (inflammasomes), to eliminate the viral infection. Recent efforts have demonstrated that inflammasomes play an important role in the clearance of viral infections (Lupfer et al., 2015). However, the mechanism by which viruses trigger the activation of the inflammasome is not yet clear.

Many types of viruses are not to be underestimated because of their variety in pathogenicity, ranging from mild reactions to the development of severe disease or even death in humans (Herrington et al., 2015). Upon recognition of the virus by the PRRs, an innate immune response is initiated, involving a cascade of signaling events. One of the most important complex proteins in innate immunity is the inflammasome. Inflammasomes are large multiprotein complexes that are assembled in response to invading pathogens and other danger signals. Inflammasomes play an important role in innate immunity and are crucial for the activation of inflammatory caspases and the subsequent processing and release of pro-inflammatory mediators (Li et al., 2021). Many viruses, such as rotavirus, Sendai virus (SeV), and Influenza A virus (IAV), can activate inflammasomes, which recruit and activate pro-caspase-1 to cleave pro-IL-1*β* and pro-IL-18, release a large amount of pro-inflammatory cytokines, cause acute or chronic inflammation, and thus clear “non-self” substances (Sharma and Kanneganti, 2016; Martinon et al., 2002). Secreted IL-1β then recruits and activates other cells in the surrounding tissue, leading to the production of chemokines, inflammatory factors, and adhesion molecules. However, not all viral invasions have an activating effect on inflammasomes. Some viruses counteract inflammasome activation and evade host immune mechanisms (Choudhury et al., 2021a; Rai et al., 2021).

## 2. Inflammasomes and their triggering inflammatory responses

The concept of “inflammasome” was first introduced by the team of Swiss scientist Jurg Tschopp, who described the mechanism of NLRP1 inflammasome formation (Martinon et al., 2002). Since then, many members of the inflammasome that function as inflammasomes have been discovered, such as NLRP3, NLRC4, AIM2, NLRP2, NLRP7, IFI16, and Pyrin. Inflammasomes are divided into two main categories: canonical inflammasomes and non-canonical inflammasomes. Canonical inflammasomes activated by caspase-1 cleave pro-IL-1*β* and pro-IL-18 to induce pyroptosis, whereas non-canonical inflammasomes activated by caspase-4/5/11 recognize lipopolysaccharide (LPS) to induce pyroptosis (Rathinam et al., 2010). In general, canonical inflammasomes are mainly composed of the sensor molecule, the adaptor protein ASC (apoptosis-associated speck-like protein containing CARD), and procaspase-1 (Lamkanfi and Dixit, 2014; Broz and Dixit, 2016). Initially, NLRs were found to serve as assembly signaling platforms to trigger NF-κB and mitogen-activated protein kinase signaling pathways and caspase-1 activation (Shaw et al., 2008); with the development of the research, AIM2 with the HIN200 structural domain was also found to form inflammasomes and cause apoptosis by acting as assembly platforms for caspase-1 activation (Rathinam et al., 2010). NLR proteins consist of three structural domains. They include the C-terminal leucine-rich repeat (LRR), which is mainly responsible for pathogen detection and recognition; the NACHT intermediate nucleotide binding and oligomerization domain, which is important for NLR oligomerization and activation; and the N-terminal protein interaction domain (PYD) or CARD, which binds to downstream linker molecules and effector proteins to initiate downstream signal transduction (Chen et al., 2021). AIM2 contains PYD domains at the N-terminus and nuclear (HIN) domains at the C-terminus. Viral dsDNA is bound to the HIN domain, and HIN-DNA binding enables AIM2 oligomerization (Wang B. et al., 2020).

Under normal conditions, NLR sensor proteins are kept in a non-activated state by LRR folding close to the NACHT, which inhibits their multimerization and prevents the assembly of inflammasomes. Once PAMPs and DAMPs are triggered, these associated antiviral genes begin to be transcribed and translated. At the same time, inflammasome sensors are activated, post-translational modifications (PTM) take place, conformational changes occur, self-repression is lost, and oligomerization and recruitment of the adapter protein ASC or pro-caspase-1 happen directly (Yang et al., 2017). PTM is an extremely important mechanism for regulating protein function, and there is considerable evidence that multiple modifications are involved in the activation of the inflammasome (Swanson et al., 2019; Xue et al., 2019). The murine E3 ubiquitin ligases can negatively regulate NLRP3 through ubiquitination (Humphries et al., 2018). TLR priming induces the phosphorylation of NLRP3 by JUN N-terminal kinase 1 (JNK1; also known as MAPK8) and promotes NLRP3 self-association and activation (Song et al., 2017). NLRP3 can be negatively regulated by sumoylation (Barry et al., 2018). Inflammasomes that form an association with ASC during inflammasome assembly are called ASC-dependent inflammasomes, such as the NLRP3 inflammasome and the AIM2 inflammasome. In addition, it has been shown that ASC is essential for NLRP1 inflammasome formation; however, this inflammasome is directly linked to pro-caspase-1 through CARD-CARD interactions (Faustin et al., 2007). The adaptor protein ASC has two structural domains: the PYD domain, which binds to PYD domain-containing sensors, and the CARD structure, which binds to CARD-containing caspase-1. Upon sensor activation, ASC, which is uniformly distributed in the cells, rapidly self-associates with dimers through a PYD-PYD interaction. It then transforms intracellularly from a soluble to an insoluble state. The ASC specks provide a platform for the recruitment and activation of caspase-1 and are therefore considered a marker of inflammasome activation (Fernandes-Alnemri et al., 2007; Prather et al., 2022). Caspase-1 is an important member of the inflammasome assembly that cleaves 34 kD pro IL-1*β* to 17 kD IL-1*β*. After ASC spot formation, procaspase-1 activates to form a caspase-1 heterotetramer. Active caspase-1 is present as a tetramer of two symmetrically arranged p20/p10 dimers (Boucher et al., 2018). In turn, pro IL-1*β* and pro IL-18 are cleaved by caspase-1 and converted into active IL-1*β* and IL-18, which are released into the cytoplasm (Thornberry et al., 1992). Simultaneously, activated caspase-1 cleaves the activated GSDMD, leading to its conversion into the cell membrane, pore formation, cell swelling, and rupture, which triggers pyroptosis in the form of inflammatory events (Shi J. et al., 2015).

**Figure 1:**
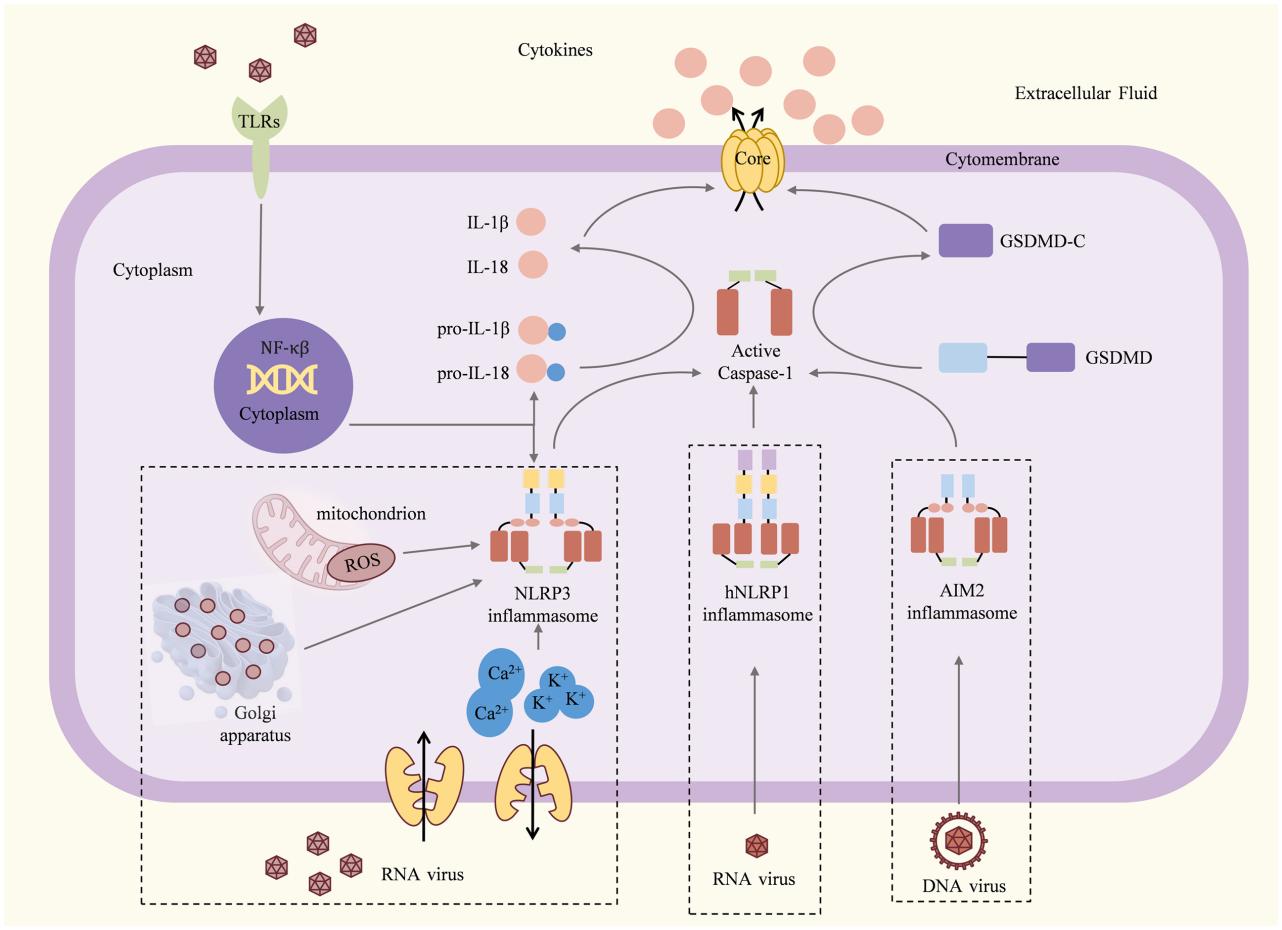
Mechanisms and consequences of inflammasome activation (Martinon et al., 2002; Shi J. et al., 2015). Once the viral particles are captured by TLRs, the associated antiviral genes begin to be transcribed and translated. For the NLRP3 inflammasome, dsRNA viruses induce K^+^ efflux, Ca2^+^ mobilization, and/or ROS or dTGN production to activate the NLRP3 inflammasome. The hNLRP1 inflammasome can be activated by RNA, viral nucleic acid, viral pathogen enzymes, and viral structural proteins. The AIM2 inflammasome can be activated by DNA or viral nucleic acid. Activated inflammasomes recruit and activate pro-caspase-1 to cleave the GSDMD, pro-IL-1*β,* and pro-IL-18. The C-terminal domain of gasdermin D can induce pyroptosis, resulting in the release of large amounts of pro-inflammatory cytokines and causing acute or chronic inflammation.

**Figure 2:**
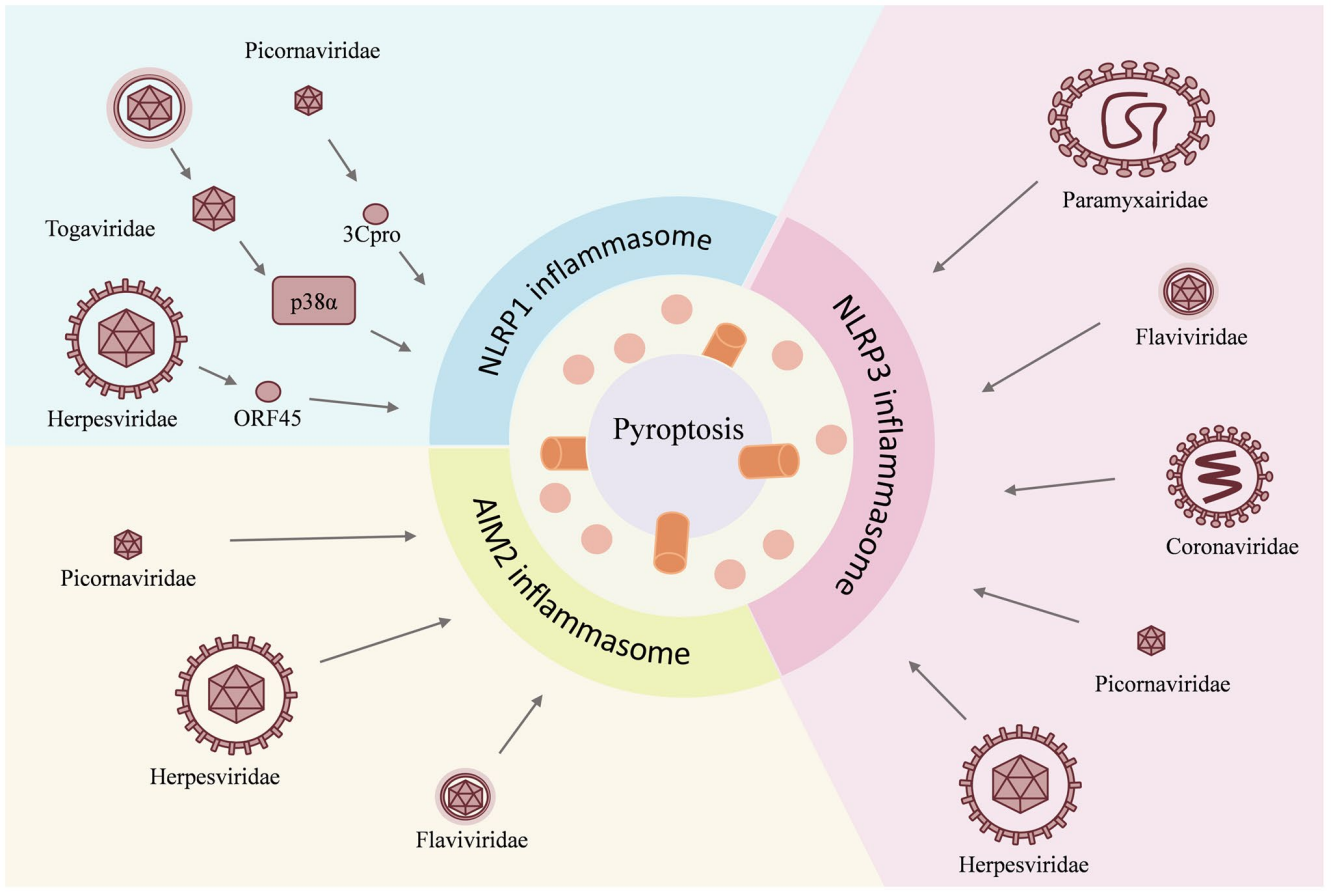
Viruses act as inducers and have different effects on individual inflammasomes. For example, the NLRP1 inflammasome is activated by the *Herpesviridae* family virus, the *Togaviridae* family virus, and the *Picornaviridae* family virus; for the NLRP3 inflammasome, the invasion of the Paramyxairidae family virus, the Flaviviridae family virus, the Coronaviridae family virus, the Picornaviridae family virus, and the Herpesviridae family virus is the cause of activation, whereas the AIM2 inflammasome is activated by the *Herpesviridae* family virus, the *Flaviridae* family virus, and the *Picornaviridae* family virus. The activation of the inflammasome then triggers the pyroptosis.

**Figure 3:**
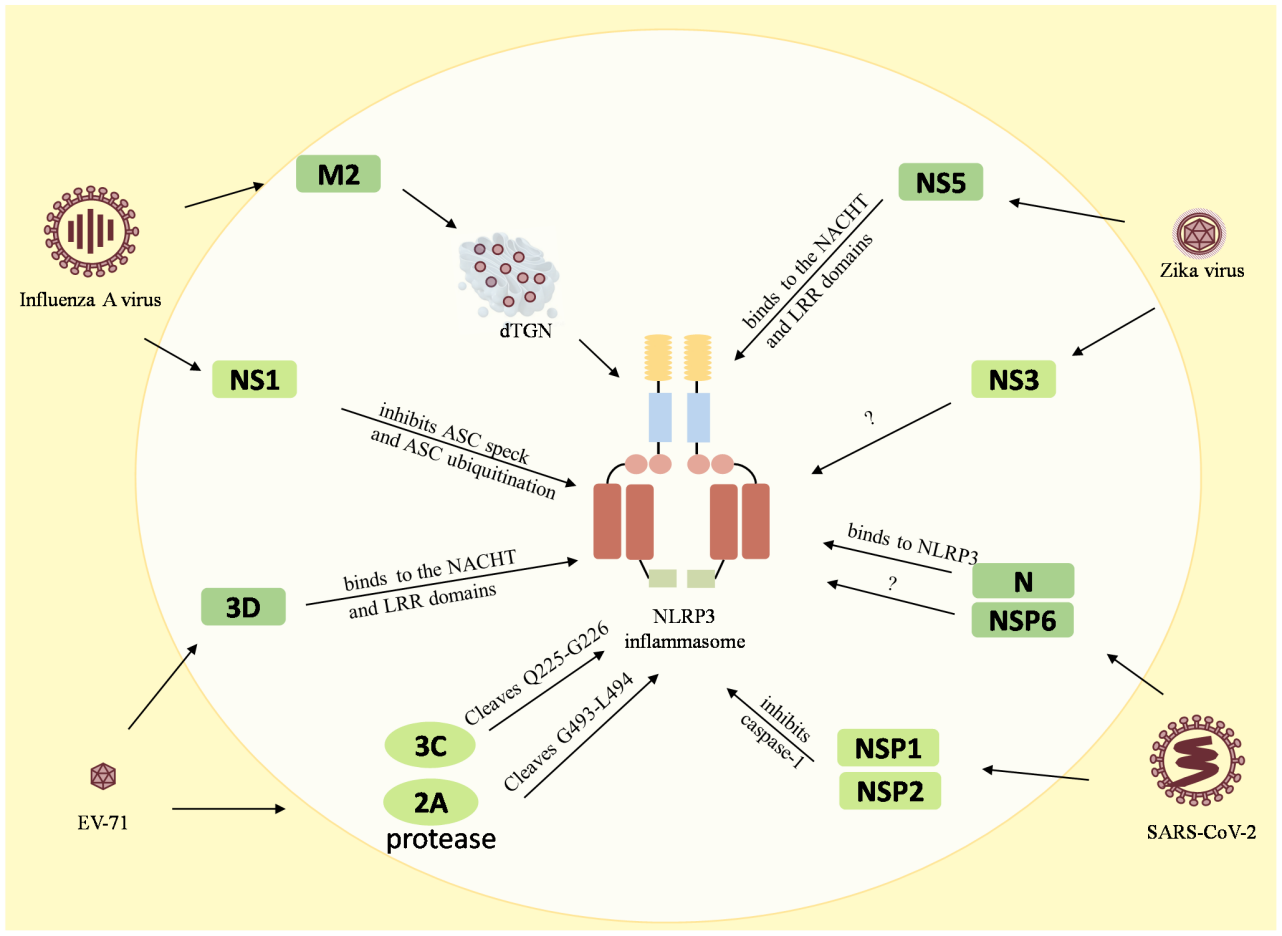
Reciprocal regulation between viruses and the NLRP3 inflammasome. The M2 protein of the influenza A virus causes dTGN dispersion to activate NLRP3 inflammasome, while the NS1 C-terminus of the pandemic H1N1 2009 virus inhibits ASC ubiquitination and thus inhibits the NLRP3 inflammasome. The enterovirus 71 3D protein binds directly to the NACHT and LRR domains of NLRP3 to activate it. However, the EV71 viral proteases 3C and 2A cleave the NLRP3 protein at the Q225-G226 and G493-L494 junctions to inhibit the NLRP3 activation inflammasome. Zika virus non-structural protein 5 (NS5) binds directly to the NACHT and LRR domains of NLRP3 to facilitate the assembly of the NLRP3 inflammasome complex, but NS3 acts directly on NLRP3 to inhibit its activation. The 260–340 aa domain of the N protein of SARSV-CoV-2 binds to NLRP3 and the NSP6 binds to ATP6API by impairing lysosomal acidification to promote NLRP3 inflammasome activation, while the NSP1 and NSP13 of SARS-CoV-2 inhibit caspase-1 activity to interfere with NLRP3 activation.

Among the various types of inflammasomes, the key inflammasomes that recognize the biological information of viruses and give a response are NLRP3, AIM2, and NLRP1 (Figures 1–3).

### 2.1. NLRP1 inflammasome

The NLRP1 inflammasome was the first type of inflammasome identified. NLRP1 is expressed in different cells in mice and humans (Fenini et al., 2020). Mice encode several NLRP1 paralogs, such as *NLRP1a*, *NLRP1b*, and *NLRP1c*. It has been proven that 129S1 *mNLRP1a* and *mNLRP1c* lack exons 1 and 2, and only *mNLRP1b* is transcribed in myeloid cells. However, human NLRP1 has only one subtype that works as an epithelial innate immune sensor in human keratinocytes and airway epithelial cells (Boyden and Dietrich, 2006; Lilue et al., 2018). Human NLRP1 is a double-stranded RNA (dsRNA) viral receptor that is activated to form the NLRP1 inflammasome, whereas mouse NLRP1 does not respond to RNA viral infection (Meyers and Zhu, 2020; Robinson et al., 2020). Human NLRP1 has a central domain (NACHT), followed by LRRs, a function-to-find domain (FIIND), and a CARD domain (Taabazuing et al., 2020). The NACHT domain, which contains Walker A and Walker B motifs, has been found to play a key role in ATP binding and ATP hydrolysis (Danot et al., 2009). The FIIND consists of the ZU5 (found in ZO-1 and UNC5) and UPA (conserved in UNC5, PIDD, and Ankyrin) subdomains. It undergoes post-translational autoproteolysis after its ZU5 subdomain is activated by the NLRP1 inflammasome (Finger et al., 2012). Upon sensor activation, the C-terminal CARD recruits pro-caspase-1 to form the inflammasome complex. Although NLRP1 inflammasome assembly does not require the involvement of ASC, its involvement with ASC enhances caspase-1 and IL-1*β* activation and release (Faustin et al., 2007). Some studies have reported that some viruses can activate NLRP1. Moreover, viral activation of NLRP1 is mediated by MAPK signaling pathways (Sandstrom, 2023).

### 2.2. NLRP3 inflammasome

NLRP3, which is highly expressed in myeloid cell types, consists of eight subdomains: the PYD (3aa-94aa) and a linker segment (95aa-130aa), the FISNA (131aa-218aa), NBD (219aa-372aa), HD1 (373aa-434aa), WHD (435aa-541aa), and HD2 (542aa-649aa) for the NACHT domain; and a transition LRR (trLRR; 650aa-742aa) and a canonical LRR (cnLRR; 743aa-1036aa) for the LRR domain (Hochheiser et al., 2022). NLRP3 interacts with the ASC via the PYD-PYD interaction. ASC then recruits pro-caspase-1 via CARD-CARD interactions to promote caspase-1 dimerization and activation. Therefore, the NLRP3-ASC-procaspase-1 complex is referred to as the NLRP3 inflammasome complex. Inactive NLRP3 forms oligomers that can assemble via LRR-LRR to form a “circular double ring” or “cage” structure, while the NACHT domains rarely interact with other domains and the PYDs are shielded inside, presumably to prevent inadvertent NLRP3 activation. Murine NLRP3 forms a dodecameric cage that shields the NLRP3 PYD to prevent premature activation, while inactive hNLRP3 forms a similar decameric structure (Hochheiser et al., 2022). The binding of NEK7, a member of the family of mammalian NIMA-related kinases (NEKs), to NLRP3 exposes its PYDs (which are capable of recruiting and nucleating ASCs) and leads to caspase-1 activation in mouse macrophages, whereas for NLRP3 in human macrophages, NEK7 appears to function in NLRP3 priming rather than activation (He et al., 2016; Sharif et al., 2019). The NLRP3 inflammasome is the best-studied inflammasome that is activated by many viral families. It has been shown that different RNA viruses from different families can activate the NLRP3 inflammasome (Choudhury et al., 2021a).

### 2.3. AIM2 inflammasome

The AIM2 inflammasome differs from NLRP1 and NLRP3 in that the sensor molecule of the AIM2 inflammasome belongs to the ALRs (Hornung et al., 2009). AIM2 has been found to bind cytosolic dsDNA (double-stranded DNA) to nucleate an inflammasome in human cell lines and murine BMDMs (Burckstummer et al., 2009; Fernandes-Alnemri et al., 2009; Hornung et al., 2009). The AIM2 is composed of a PYD at the N-terminal and a HIN domain at the C-terminal. AIM2 binds to ASC through a PYD-PYD interaction, while ASC binds to procaspase-1 through a CARD-CARD interaction, forming the AIM2-ASC-procaspase-1 complex (Kumari et al., 2020). As shown by electron microscopy images, the AIM2 inflammasome is a ternary complex centered on the adaptor protein ASC. The spatial structure of the HIN200 structural domain of AIM2 binds to dsDNA by electrostatic interaction, and the same dsDNA binds to multiple AIM2 molecules and aggregates to form an inflammasome (Roberts et al., 2009). The AIM2 inflammasome has been shown to be activated by DNA viruses, so AIM2 induces pyroptosis to eliminate virus-infected cells (Rathinam et al., 2010).

## 3. Modulation of inflammasome activation by viral infection

Viral infections can cause mild or life-threatening diseases, so it is important to understand viral pathogenesis and disease pathogenesis (Rai et al., 2021). The major components of viruses are nucleic acids and proteins, which are used as inducers to activate inflammasomes (Spel and Martinon, 2021). Activation of the inflammasome triggers pyroptosis, which induces inflammation in the host. However, inflammasome signaling can be both protective (pathogen clearance) and pathogenic (cytokine storm) in viral diseases. In order to sustain infection, viruses gradually evolve to inhibit the activation of inflammasomes. Understanding these mechanisms of viral activation and/or inhibition may provide new insights to improve host antiviral immunity.

### 3.1. Viruses act as inducers that activate the inflammasome

The process of viral infection is accompanied by the development of an inflammatory response. Recognition of the different components of the virus leads to the activation of the inflammasome and consequently to the occurrence of a cytokine storm. Some viruses activate a variety of inflammasomes through different pathways and cause inflammation. Among the virus-associated inflammasomes, NLRP1 primarily recognizes dsRNA viruses, NLRP3 primarily recognizes ssRNA (single-stranded RNA) viruses, and AIM2 primarily recognizes DNA viruses. These viruses act as inducers with different effects on individual inflammasomes.

#### 3.1.1. Activation of the NLRP1 inflammasome by viral infections

Activation of The LRP1 inflammasome is not only initiated with the recognition of viral nucleic acid; viral pathogenic enzymes and structural proteins can also cause the activation of the NLRP1 inflammasome [39]. It was found that NLRP1 forms a complex structure with dipeptidyl peptidase 9 (DPP9), which inhibits the activation of NLRP1. On the other hand, the inhibitor VbP inhibits the formation of this complex and facilitates the activation of NLRP1 (Zhong et al., 2018). Sand et al. (2018) found that some viruses act on the protease or structural protein of human NLRP1, triggering human NLRP1 activation. With the advancement of NLRP1 research, the mechanism of NLRP1 activation by various pathogens has become evident, which would be helpful for the future treatment of NLRP1-related diseases.

##### 3.1.1.1. *Togaviridae* family

The *Togaviridae* are (+) ssRNA viruses that are transmitted to vertebrate hosts primarily by mosquitoes and are referred to as arthropod-borne viruses or arboviruses (Westcott et al., 2023). NLRP1 activation by some alphaviruses, such as Semliki Forest virus (SFV), *Solenopsis invicta* virus (SINV), Ross River virus (RRV), Mayaro virus (MAYV), and Chikungunya virus (CHIKV), was found to be dependent on p38 kinase activity (Jenster et al., 2023). Bauernfried et al. (2021) reported that long dsRNA generated during Semliki Forest virus infection binds and activates NLRP1 in human epithelial cells. When PYD recognizes dsRNA, LRR binds to dsRNA and causes protein conformational changes through NACHT hydrolysis of ATP to activate IL-1*β*, ASC, and caspase-1-dependent inflammasome assembly (Chui et al., 2019). However, the required length of dsRNA is longer than 500 bp, and the activated NLRP1 inflammasomes have been found to be more stable with increasing viral nucleic acid length (Bauernfried et al., 2021).

##### 3.1.1.2. *Picornaviridae* family

The genus Enterovirus and the genus *Rhinovirus* are (+) ssRNA viruses in the family *Picornaviridae*. The most common species infecting humans are Coxsackievirus B3 (CVB3), Enterovirus D68 (EV68), Poliovirus 1 (PV1), and Human Rhinovirus A (HRV) (Tsu et al., 2021). *Picornaviruses* cleave residues Q130-G131 of NLRP1 via their 3C pro-proteins through the N-terminal glycine degron pathway to cleave the N-terminal fragment, thereby releasing the active C-terminus for NLRP1 inflammasome assembly (Robinson et al., 2020). These viruses activate NLRP1 by direct proteolytic cleavage of the N-terminus by viral proteases (Tsu et al., 2021). When NLRP1 is degraded by the proteasome, the self-hydrolysis site (F983-S984) within the FIIND domain is hydrolyzed, releasing the new domain to form a new NLPR1 fragment, such as the UPA sub-structural domain and the CARD domain (D’Osualdo et al., 2011). The NLRP1 C-terminus does not enter the proteasome with the full-length protein but completes self-assembly and recruits caspase-1 through the CARD domain to achieve NLRP1 inflammasome assembly (Zhong et al., 2018).

##### 3.1.1.3. *Herpesviridae* family

The dsDNA virus *Herpesviridae* family is divided into three sub-families: the *alpha herpesvirus*, *the beta herpesvirus,* and t*he gamma herpesvirus*. Kaposi’s sarcoma, associated with the *gamma herpesvirus* (KSHV), causes an inflammatory and angiogenic endothelial cell neoplasm (Polizzotto, 2023). KSHV has been shown to activate the NLRP1 inflammasome with its structural protein ORF45. However, this pathway is not part of the classical functional degradation achieved by the degradation mechanism (Sandstrom et al., 2019). Normally, a Linker1 region between the PYD and NACHT domains inhibits NLRP1 activation by binding to the C-terminal UPA domain. However, upon recognition of viral infection, the ORF45 structural protein of KSHV binds to Linker1, inhibiting NLRP1 activation and inflammasome assembly (Yang et al., 2022).

#### 3.1.2. Activation of the NLRP3 inflammasome by viral infection

Compared to other inflammasomes, NLRP3 is the one that is most extensively studied. The known classical NLRP3 pyroptosis pathway is that the pathogens trigger the activation and assembly of NLRP3 inflammasome and release of caspase-1, which cleaves GSDMD and triggers cell pyroptosis. In addition, overexpression of NLRP3 has been found to cause great damage and even threaten the life of the host. Therefore, NLRP3 has become a very popular therapeutic target with the potential to treat a variety of inflammatory diseases, including autoimmune diseases (Sutterwala et al., 2006) and metabolic disorders (Zhou et al., 2010). Furthermore, some researchers have tried to verify the feasibility of using NLRP3 as a therapeutic target for COVID-19 (Yang et al., 2019). In antiviral innate immunity, NLRP3 recognizes a variety of PAMPs and DAMPs generated during viral replication, which trigger the NLRP3 inflammasome-dependent antiviral immune responses and facilitate viral suppression. Thus, NLRP3 is considered to be one of the most important inflammasomes against viral infection and contributes greatly to the stimulation of the host’s natural antiviral immunity.

The possible factors for the activation mechanism of the NLRP3 inflammasome are mainly altered ion fluxes, reactive oxygen species (ROS) production, lysosomal destabilization, and post-translational modification of NLRP3 (Yang et al., 2019). A recent study has shown that the dispersed trans-Golgi network (dTGN) is also an important factor in triggering NLRP3 inflammasome activation (Chen and Chen, 2018). Some viral invasion processes have proteins that can directly act on NLRP3, which in turn activates NLRP3 inflammasome formation and ultimately triggers cell pyroptosis to inhibit viral replication.

##### 3.1.2.1. *Paramyxairidae* family

It has been reported that some viruses belonging to the (+) ssRNA (single-stranded RNA) virus family *Paramyxairidae* are capable of activating the NLRP3 inflammasome, such as Newcastle disease virus (NDV), human metapneumovirus (HMPV), respiratory syncytial virus (RSV), and Peste Des Petits Ruminants Virus (PPRV) (Table 1). NDV leads to acute fever and sepsis in poultry. Infection with virulent strains of NDV causes a strong immune response, resulting in the release of large quantities of inflammatory factors such as IL-1*β*, IL-6, IL-18, and IFN-*β* (Hu et al., 2015; Li et al., 2016; Cai et al., 2023). Wang et al. (2016) found that NDV infection activated the NLRP3 inflammasome in PMA-stimulated THP-1 cells. However, the mechanism by which inflammasome components recognize NDV is unclear. Gao et al. (2020) demonstrated that the NDV RNA induced IL-1*β* expression via the NLRP3/caspase-1 inflammasome in DF-1 cells. However, there is no effective evidence to prove which components of NDV viral RNA are required for the induction of IL-1*β*. Similarly, the effects of NDV non-structural proteins in addition to the RNA also need to be confirmed. Human metapneumovirus (HMPV) was first isolated from young children in the Netherlands in 2001, and it is now recognized as a leading cause of respiratory tract infections in infants (Van den Hoogen et al., 2001; Wang et al., 2021). It is reported that with the increased production of IL-1*β* and IL-18, NLRP3 mRNA expression was upregulated in HMPV-infected children as compared with the healthy control subjects (Malmo et al., 2016). However, the authors did not clarify the role of the NLRP3 inflammasome during HMPV infection. Le et al. (2019) found for the first time that both NLRP3 inflammasome inhibition and SH protein deletion attenuated inflammation and lung injury and concluded that NLRP3 inflammasome activation is triggered by the viral SH protein during HMPV infection.

**Table 1:** Summary of viral components that activate the nlrp3 inflammasome.

Virus family	Virus	Activator	Possible mechanism	References
*Paramyxairidae* family	NDV	RNA	-	Gao et al. (2020)
	HMPV	RNA and 2B	ion efflux (Ca^2+^ efflux)	Zhi et al. (2020)
	RSV	-	ROS	Bedient et al. (2020)
	PPRV	N protein	binding to MyD88	Li et al. (2022)
*Flaviviridae* family	DENV	M Protein	interacts with NLRP3	Pan et al. (2019)
		EIII	-	Lien et al. (2021)
	DENV-2	2A and 2B proteins	ROS	Shrivastava et al. (2020)
	HCV	-	deubiquitination by UCHL5	Ramachandran et al. (2021)
		core protein	--	Negash et al. (2019)
	ZIKV	NS5 protein	interacts with NLRP3	Wang et al. (2018)
		NS1 protein	cleavage cGAS	Zheng et al. (2018)
	CSFV	-	-	Fan et al. (2018)
	HBV	X protein	-	Xie et al. (2020)
*Picornaviridae* family	FMDV	Lpro	interacted with NLRP3	Choudhury et al. (2021b)
		RNA and 2B	ion efflux (Ca2+ efflux)	Zhi et al. (2020)
	ECHO11	2B protein	interacts with NLRP3	Wang et al. (2022)
	EV71	3D protein	interacts with NLRP3	Wang et al. (2017)
	SVA	3D protein	interacts with NLRP3	Choudhury et al. (2022)
	EMCV	2B protein	ion efflux (Ca2+ efflux)	Ito et al. (2012)
*Coronaviridae* family	SARS-CoV	N protein	-	Pan et al. (2021a)
		3A protein	K efflux and ROS production	Chen et al. (2019)
		ORF3a protein	promoting TRAF3-mediated ASC ubiquitination	Siu et al. (2019)
	SARS-CoV-2	SH protein	-	Le et al. (2019)
*Herpesviridae* family	HSV-1	DNA	STING	Wang W. et al. (2020)
*Orthomyxoviridae* family	HAV	M2 protein	dTGN	Pandey and Zhou (2022)
*Alphaviridae* family	MAYV	-	potassium efflux and ROS production	De Castro-Jorge et al. (2019)
*Filoviridae* family	EboV	-	-	Halfmann et al. (2018)
*Arteriviridae* family	PRRSV	Replication-transcription complex (RTC)	dTGN	He et al. (2022)
*Rhabdoviridae* family	RV	-	-	Lawrence et al. (2013)
*Bunyaviridae* family	SFTSV	-	-	Liu et al. (2021)
	RVFV	-	-	Ermler et al. (2014)

NDV, Newcastle disease virus; HMPV, Human metapneumovirus; RSV, Respiratory syncytial virus; PPRV, Peste Des Petits Ruminants Virus; DENV, Dengue Virus; HCV, Hepatitis C virus; ZIKV, Zika virus; CSFV, Classical Swine Fever Virus; HBV, Hepatitis B virus; FMDV, Foot-and-mouth disease virus; ECHO11, Echovirus 11; EV71, Enterovirus 71; SVA, Senecavirus; EMCV, Encephalomyocarditis Virus; SARS-CoV, Severe acute respiratory syndrome coronavirus; SARS-CoV-2, Severe acute respiratory syndrome coronavirus-2; HSV-1, Herpes Simplex Virus 1; HAV, Influenza A Virus; MAYV, Mayaro virus; EBoV, Ebola virus; PRRSV, Porcine Reproductive and Respiratory Syndrome Virus; RV, Rabies Virus; SFTSV, Severe fever with thrombocytopenia syndrome virus; RVFV, Rift Valley fever virus.

##### 3.1.2.2. *Flaviviridae* family

The (+) ssRNA virus family *Flaviviridae* consists of three genera: *flavivirus*, *pestivirus,* and *hepacivirus*. Viral proteins from the *flaviviruses* Dengue and Zika and the hepatitis B and C *hepaciviruses* can activate the NLRP3 inflammasome. This section describes how the Dengue virus and Hepatitis C virus can do so.

Dengue virus (DENV) is an enveloped virus with positive-sense ssRNA that causes severe clinical symptoms such as fever, myalgia, arthralgia, headache, and even dengue hemorrhagic fever (DHF) if untreated (Azhar et al., 2015). The virion surface envelope protein domain III (EIII) of DENV can induce cell death in endothelial cells, neutrophils, and megakaryocytes. Lien et al. found that EIII-induced thrombocytopenia, platelet activation, and cell death were significantly ameliorated by blocking EIII-induced platelet signaling using the competitive inhibitor chondroitin sulfate B or the selective NLRP3 inflammasome inhibitors OLT1177 and Z-WHED-FMK (Lien et al., 2021). The amphipathic helix is part of the M protein, which interacts with envelope protein (E) to promote DENV invasion, packaging, and release. During DENV infection, the M protein directly interacts with NLRP3 through its NBD and LRR domains to promote the assembly of the inflammasome complex (Pan et al., 2019). Several non-structural proteins of DENV have also been shown to be associated with the NLRP3 inflammasome expansion. Shrivastava et al. showed that DENV-2 NS2B and DENV-2 NS2A (which act as viroporins via calcium influx) and ROS production trigger the inflammasome and high levels of IL-1*β* in HMEC-1 as well as THP-1 and HepG2 via NLRP3 inflammasome activation. Besides the proteins of DENV discussed above, other proteins, such as NS1 (a viral protein secreted by infected cells), have been found to play an important role in the inflammasome under DNEV infection (Quirino-Teixeira et al., 2020). However, the mechanism of inflammasome activation by these proteins is still unclear (Quirino-Teixeira et al., 2020).

Hepatitis C virus (HCV) infection remains a major cause of hepatic inflammation and liver disease. Initial infection with HCV is sufficient to cause chronic inflammatory hepatitis, which may increase the possibility of liver tumorigenesis (Zheng et al., 2017). The process of NLRP3 activation by HCV involves multiple post-translational modification processes, n phosphorylation, ubiquitination, and de-ubiquitination (Seok et al., 2020). Several PTM regulators have been identified that act on individual amino acids of NLRP3 to regulate NLRP3 inflammasome activation. It has been found that chronic hepatitis C virus (HCV) and herpes simplex virus (HSV) act as viral “activators” of the PTM of NLRP3 (Siu et al., 2019; Wang W. et al., 2020). After HCV infection, Recombinant Ubiquitin Carboxyl Terminal Hydrolase (UCHL5) mediates the de-ubiquitination of NLRP3 (Wang W. et al., 2020), while the stimulator of interferon genes (STING), which functions as an NLRP3 activator, interacts with NLRP3 and then inhibits the ubiquitination of NLRP3 (Siu et al., 2019). Chen et al. identified that HCV RNA could activate the NLRP3 inflammasome through the involvement of ROS production. Negash et al. (2019) indicated that activation of the NLRP3 inflammasome by the HCV core protein requires calcium mobilization (Wallace et al., 2022).

##### 3.1.2.3. *Picornaviridae* family

*Picornaviridae* is a family of viruses that can cause a wide array of diseases in humans and animals, resulting in large economic losses. It comprises five genera: *Enterovirus*, *Hepatovirus*, *Rhinovirus*, *Apthovirus*, and *Cardiovirus*. *Enterovirus*, *Hepatovirus,* and *Rhinovirus* mainly infect humans; *Apthovirus* (referred to as the foot-and-mouth disease virus) infects cloven-hoofed animals and occasionally humans; and *Cardioviruses* infect rodents (Zell et al., 2017). Previous studies have shown that Picornaviridae viruses, such as foot-and-mouth disease virus (FMDV), encephalomyocarditis virus (EMCV), and enterovirus 71 (EV71), induce NLRP3 inflammasome activation. Foot-and-mouth disease virus (FMDV), a member of the *Picornaviridae* family, causes foot-and-mouth disease (FMD) in domestic cloven-hoofed animals; it is characterized by fever, lameness, and vesicular lesions (Grubman and Baxt, 2004). Some studies have shown that FMDV RNA, 2B protein, and L pro activate the NLRP3 inflammasome by inducing IL-1*β* production. FMDV RNA induces NF-κB activation, upregulates the transcription of NLRP3 and pro-IL-1*β*, triggers the NLRP3 inflammasome, and produces IL-1*β* expression in macrophages. FMDV 2B activates the NLRP3 inflammasome through the elevation of intracellular ions and induces IL-1*β* dependent on the helical transmembrane region (Zhi et al., 2020). FMDV L pro interacts with the NACHT and LRR domains of NLRP3 to promote NLRP3-ASC assembly and IL-1*β* production and induces calcium influx and potassium efflux to activate the NLRP3 inflammasome (Choudhury et al., 2022).

The proteins involved in the different genera of *Picornaviridae* are also found to induce inflammatory responses. For example, Echovirus 11 (ECHO 11) 2B interacts with NLRP3 to facilitate inflammasome assembly (Wang et al., 2022); EV71 3D can interact with the NACHT and LRR domains of NLRP3, which regulate NLRP3 inflammasome assembly (Wang et al., 2017); Senecavirus 3D interacts with the NACHT domain of NLRP3 to induce IL-1*β* production via the NF-κB signaling pathway and ion channel signaling (Choudhury et al., 2022); and EMCV viroporin 2B is involved in Ca^2+^ influx, which activates the NLRP3 inflammasome (Ito et al., 2012).

##### 3.1.2.4. *Coronaviridae* family

*Coronaviridae* are (+) ssRNA viruses that are characterized by gastrointestinal and respiratory disease in humans, poultry, and bovines (Siddell et al., 1983). In humans, a strain known as the Severe acute respiratory syndrome coronavirus(SARS coronavirus)causes a highly contagious respiratory disease that is characterized by fever, cough, muscle aches, and difficulty breathing. SARS-CoV proteins play an important role in inflammation. The viroporin E forms Ca^2+^-permeable ion channels and activates the NLRP3 inflammasome (Nieto-Torres et al., 2015). The 3a protein induces intracellular K^+^ efflux and causes mitochondrial damage, thereby activating the NLRP3 inflammasome (Chen et al., 2019). ORF3a protein activates the NLRP3 inflammasome by promoting TRAF3-dependent ubiquitination of ASCs (Siu et al., 2019).

In late 2019, a virus closely related to the SARS coronavirus emerged in Wuhan, China (Anka et al., 2021). The virus, later named severe acute respiratory syndrome coronavirus 2 (SARS-CoV-2), caused an illness known as COVID-19, which was similar to SARS and was primarily characterized by fever and respiratory symptoms (Polak et al., 2020). SARS-CoV-2 triggers inflammasome-dependent release of the pro-inflammatory cytokine IL-1β and pyroptosis (Aymonnier et al., 2022). The N protein directly interacts with the NLRP3 protein, which promotes NLRP3 binding to ASC and facilitates NLRP3 inflammasome assembly (Pan et al., 2021b). However, the mechanism of SARS-Cov2 on NLRP3 inflammasome activation is still unclear and needs to be further explored.

##### 3.1.2.5. *Herpesviridae* family

*Herpesviridae is a* family of enveloped, dsDNA viruses with relatively large, complex genomes. It has been found that members of the *Herpesviridae* family, such as Herpes simplex virus 1, infect a wide range of vertebrates, namely mammals, birds, and reptiles. Herpes simplex virus 1 (HSV-1) activates the NLRP3 inflammasome differently compared with other viruses. Hu et al. (2022) found that the inflammasome and GSDMD-mediated pyroptosis exert great effects on the process of HSV-1 infection. Moreover, it was found that HSV-1 causes macrophage pyroptosis by inducing NLRP3 inflammasome activation, cleavage of caspase-1 and gasdermin D, and increasing IL-1*β* production. However, the mechanism of HSV-1 on the NLRP3 inflammasome activation still needs to be explored (Hu et al., 2022).

#### 3.1.3. Activation of the AIM2 inflammasome by viral infection

AIM2 has previously been shown to act as a DNA sensor for intrinsic immunity. However, AIM2 is currently used to detect bacterial DNA sources. Therefore, it has been suggested that AIM2 is associated with inflammation caused by partial viral invasion infections (Fernandes-Alnemri et al., 2009). The HIN structural domain of AIM2 is mainly composed of double OBs (OB1 and OB2) that are folded and connected, suggesting that AIM2 recognizes dsDNA (Roberts et al., 2009). Upon relevant viral invasion, the HIN domain of AIM2 binds to viral DNA through electrostatic interaction, and the PYD structural domain binds to the ASC, which recruits caspase-1 and forms the AIM2 inflammasome, thereby promoting the production of IL-1*β* and IL-18 or triggering cell pyroptosis (Hornung et al., 2009). However, aberrant activation of AIM2 can cause immune-related diseases such as systemic lupus erythematosus (SLE) (Panchanathan et al., 2010), psoriasis (Dombrowski et al., 2011), primary Sjögren’s syndrome (Vakrakou et al., 2020), and arthritis (Baum et al., 2015). Notably, activation of AIM2 pairs is induced by cytoplasmic dsDNA and is not triggered by binding of specific sequences. However, the ability of dsDNA to activate AIM2 depends mainly on its length, with the minimum ability to activate AIM2 at 70 bp, while the best ability can be observed at 200 bp (Morrone et al., 2015).

##### 3.1.3.1. *Herpesviridae* family

Some *Herpesviridae* viruses can activate the AIM2 inflammasome, IL-18, and IL-1*β*. Members of the subfamily *beta herpesviridae* with host specificity for mice can produce latent infection of immunocytes and lead to inflammatory responses (Ikuta et al., 2015). Recent studies have shown that the MCMV-infected cochlea displays a robust and chronic inflammatory response with impairment of the sensorineural hearing system (Schachtele et al., 2011). Inflammasome-related kinase caspase-1 and downstream inflammatory factors IL-1*β*, IL-18, and AIM2 protein were increased and activated after CMV infection in the cochlea (Shi X. et al., 2015). These studies provided evidence that the AIM2 inflammasome plays a key role in MCMV-infected inflammation. Therefore, the AIM2 inflammasome may be a novel target for the prevention and treatment of CMV-related SNHL.

Normally, AIM2 acts as a DNA sensor in the host. However, recent studies have shown that some RNA viruses also induce increased expression of AIM2. Hamel et al. found that infection with Zika virus (ZIKV, a member of the *Flaviviridae* family, causes a mosquito-mediated arthropod-borne disease in humans) increased the expression of AIM2 and IL-1*β* in skin fibroblasts (Hamel et al., 2015). Enterovirus A71 (EV-A71) is a human RNA virus belonging to the family *Picornaviridae* that causes sporadic and epidemic hand, foot, and mouth disease (HFMD) in young children aged 5 and younger (Wang et al., 2014). In EV-A71 infected SK-N-SH and SK-N-SH/si AIM2 cells, the co-localization of virus and AIM2 antigens was found, suggesting AIM2 inflammasome-induced pyroptosis following EV-A71 infection (Yogarajah et al., 2017).

To date, we have only been able to demonstrate that these viral infections induce the expression of AIM2 and its downstream factors, but there are few studies on the specific mechanisms underlying AIM2 activation. Therefore, there is a potential need for studies on diseases triggered by elevated AIM2 activation.

### 3.2. Viruses act as inhibitors to suppress inflammasome activation

Several viruses are capable of inhibiting the inflammasome to escape the antiviral immunity in the process of infecting the host. Therefore, we expound on certain viruses that can inhibit the activation of the inflammasome, which would be valuable for relevant researchers who highly desire the search for inflammation inhibitors.

#### 3.2.1. NLRP1 inflammasome inhibition

NLRP1 is mainly expressed in neurons and microglia in brain tissue. Excessive activation of NLRP1 triggers central nervous system-related disorders such as cerebral hemorrhage, Alzheimer’s disease, ischemic stroke, and epilepsy. Under the conditions of these diseases, a significantly increased NLRP1 inflammasome has been found. However, with the use of partial inhibitory drugs, the expression of IL-1*β* and IL-18 is reduced, thereby decreasing the activation of the inflammasome. Under normal conditions, NLRP1 is in a stable state due to its self-inhibitory structure. However, with the invasion of external factors, NLRP1 inflammasome activation is triggered and causes pyroptosis.

In addition to the autoinhibitory structure of NLRP1, there are some viruses that inhibit the NLRP1 activation to enhance their virulence factors and evade the host immune mechanism. For example, the cowpox F1L protein is a cytosolic Bcl-2 viral homolog with apoptosis-inhibiting effects (Gerlic et al., 2013). Additionally, an inflammatory response to Vaccinia virus (VACV) hosts was observed in mice infected with F1L-deficient viruses, and the F1L protein was found to inhibit NLRP1 activation mainly through its residues 32–37 (Gerlic et al., 2013). However, these results focus mainly on murine NLRP1 and not on human NLRP1. Many other viruses capable of inhibiting NLRP1 activation remain to be explored.

#### 3.2.2. NLRP3 inflammasome inhibition

Activation of the NLRP3 inflammasome forms and releases a large number of pro-inflammatory factors that play an important role in inflammation. Activation of the inflammasome eventually triggers pyroptosis, a process that is not conducive to viral transmission in the host or longevity in nature. However, some viruses evade host immune mechanisms along with NLRP3 inflammasome activation. Influenza A virus can antagonize NLRP3 inflammasome activation by blocking post-translational modifications of ASC by the NS1 protein, thereby preventing inflammasome assembly (Moriyama et al., 2016). Sendai virus inhibits NLRP3 inflammasome activation through direct interaction between the V protein and the NLRP3 protein (Komatsu et al., 2018). The measles virus V protein also interacts with the NLRP3 protein to prevent inflammasome activation (Komune et al., 2011). Interaction between the NLRP3 and human parainfluenza virus type 3\u00B0C protein also prevents inflammasome assembly and activation (Shil et al., 2018). Adenovirus (Ad5) VA RNAi inhibits NLRP3 inflammasome activation by targeting the cellular proinflammatory protein PKR (Darweesh et al., 2019). This evidence suggests that viruses can directly antagonize NLRP3 inflammasome activation through their proteins and/or nucleic acids to escape the host’s natural antiviral immunity.

#### 3.2.3. AIM2 inflammasome inhibition

It is well established that viral infections trigger activation of the AIM2 inflammasome. However, some studies have found that some viral proteins inhibit AIM2 inflammasome assembly and evade the host’s natural immune response. For example, the VP22 protein of HSV interacts with AIM2 and disrupts its oligomerization, thereby inhibiting AIM2 inflammasome activation (Maruzuru et al., 2018). In HCMV-infected THP-1 cells, pUL83 of HCMV binds to AIM2, resulting in the inhibition of AIM2 activation along with down-regulation of the expression of downstream molecules (Huang et al., 2017). However, to our knowledge, only a few studies have discovered the inhibition of the AIM2 inflammasome by viral proteins. Therefore, other viruses with the same function still need to be discovered and studied in depth to provide new targets for the treatment of diseases caused by the overexpression of AIM2.

## 4. Reciprocal regulation between virus and NLRP3 inflammasome

Many viruses have been shown to reciprocally regulate the inflammasome, especially the NLRP3 inflammasome. Influenza A virus (Allen et al., 2009), enterovirus 71 (EV-71), Zika virus, and SARS-CoV-2, have been found to interact with both activation and inhibition of the NLRP3 inflammasome. The influenza A virus M2 protein causes dTGN dispersion through its ion channel function, which recruits NLRP3 to the dTGN to oligomerize and thereby recruits ASC to form the NLRP3 inflammasome (Ichinohe et al., 2010; Pandey and Zhou, 2022). PB1-F2 proteins derived from IAV strains activate the NLRP3 inflammasome and induce lung inflammation, thus demonstrating NLRP3-dependent cellular recruitment (McAuley et al., 2013; Pinar et al., 2017). On the other hand, it was found that the NS1 C-terminus of the 2009 pandemic H1N1 acts on target lysine residues, K110 and K140, which inhibit ASC ubiquitination and in turn disrupt NLRP3 inflammasome-mediated IL-1*β* production (Park et al., 2018). The enterovirus 71 3D protein (an RNA-dependent RNA polymerase) directly binds to the NACHT and LRR domains of NLRP3 to facilitate the assembly of the inflammasome complex by forming a 3D-NLRP3-ASC ring-like structure (Wang et al., 2017). Meanwhile, the EV71 viral proteases 3C and 2A cleave the NLRP3 protein at the Q225-G226 and G493-L494 junctions and inhibit NLRP3 inflammasome activation (Wang et al., 2015). In addition, 3C also directly cleaves GSDME at the Q193-G194 pair, resulting in a downstream molecule of the NLRP3 inflammasome, inhibition of the cell pyroptosis, and evasion of the antiviral responses (Lei et al., 2017). Zika virus non-structural protein 5 (NS5) directly binds to the NACHT and LRR domains of NLRP3 to promote ASC oligomerization and form NS5- NLRP3-ASC sphere-like structures, which facilitate the assembly of the NLRP3 inflammasome complex (Wang et al., 2018). Conversely, NS3 (a serine protease) acts directly on NLRP3 and inhibits its activation (Gim et al., 2019). In addition, the nucleocapsid (N) protein and the non-structural protein NSP6 of SARS-CoV-2 are capable of activating the NLRP3 inflammasome. The domain containing amino acids (aa) 260aa–340aa of the N protein binds to NLRP3 to promote ASC oligomerization and activate NLRP3 inflammasome assembly (Pan et al., 2021a). The NSP6 binds to ATP6API by impairing lysosomal acidification, which in turn promotes NLRP3 inflammasome activation (Sun et al., 2022). Meanwhile, some of the nonstructural proteins, namely NSP1 and NSP13 of SARS-CoV-2, have been shown to interfere with NLRP3 activation by inhibiting caspase-1 activity (Kim et al., 2021).

It is not surprising that viruses are recognized by the host’s innate immune system and have mechanisms associated with the suppression of host immunity. As foreign invaders, viruses are also recognized by relevant structures in the host that respond by eliminating the “foreigners.” However, viruses evade the relevant aspects of the host immune machinery to replicate and proliferate on their own. During the infectious phase of SARS-CoV-2, early viral products (e.g., NSP1 and NSP13) exert an inhibitory effect on NLRP3 inflammasome activation to facilitate viral replication and assembly, whereas late viral products (e.g., N and ORF3a) motivate NLRP3 inflammasome-mediated pyroptosis, promoting viral spread and pathogenesis (Nieto-Torres et al., 2014). A clear understanding of the mechanism by which viruses activate and inhibit inflammasomes will enable precise and effective prevention and treatment of some viral diseases that are currently difficult to treat.

## 5. Conclusion

Inflammation is a protective response caused by damage to the organism that effectively removes harmful factors and damaged tissues and facilitates the organism’s recovery. Inflammatory phenomena are prevalent in various pathological changes and are mediated by a series of endogenous and exogenous inflammatory mediators. Inflammatory factors that cause tissue damage and induce an inflammatory response, bind to relevant receptors in the body and subsequently induce the release of pro-inflammatory cytokines. Pro-inflammatory cytokines then activate many types of immune cells and promote the inflammatory response. Infections caused by viruses trigger inflammatory responses to varying degrees. Inflammasomes play an important role in inflammation triggered by viral infections. Once the viral invasion of host cells is recognized by relevant receptors, inflammasome assembly is induced along with the activation of cell death-associated proteins and the prevention of viral parasitism in host cells. Cell death is primarily triggered by the activation of caspase-family-related proteins that cleave the GSDM family, resulting in cell pyroptosis. Upon activation of cell pyroptosis, the cytosolic contents are released, where activated pro-inflammatory factors act on the associated immune cells to activate the inflammatory response and clear the foreign particles. Pyroptosis is also a double-edged sword. Mild pyroptosis facilitates the enhancement of host immunity, while excessive cell pyroptosis can cause serious disease in the organism. However, the limitations of current experiments, such as the use of animal models, cell lines, or *in vitro* systems, may not fully reflect the human situation.

Currently, some compounds have been found to have the function of inhibiting the inflammasome (mostly NLRP3), which provides a clear target and idea to inhibit inflammation triggered by viral infection. However, the activation of the inflammasome by viruses is not completely consistent, and some viruses possess both activation and inhibition properties in the inflammasome. Therefore, this review has identified novel inflammasome sensors or regulators, elucidated the molecular mechanisms of inflammasome activation or inhibition by viruses, explored the crosstalk between inflammasome and other immune pathways, and developed novel inflammasome-based antiviral strategies.

## Author contributions

WS and MJ wrote the original draft. HC, LY, SL, XW, ZW, FM, and FE revised and edited the manuscript. HS and CO supervised the manuscript. All authors read and approved the published version of the manuscript.

## Funding

The project is supported by the National Natural Science Foundation of China (32260876), Guangxi Science and Technology Base and Talent Special (2021 AC19372), and scientific research startup funds of Guangxi University.

## Conflict of interest

ZW was employed by QYH Biotech.

The remaining authors declare that the research was conducted in the absence of any commercial or financial relationships that could be construed as a potential conflict of interest.

## Publisher’s note

All claims expressed in this article are solely those of the authors and do not necessarily represent those of their affiliated organizations, or those of the publisher, the editors and the reviewers. Any product that may be evaluated in this article, or claim that may be made by its manufacturer, is not guaranteed or endorsed by the publisher.
